# State-Specific Prevalence and Characteristics of Frequent Mental Distress and History of Depression Diagnosis Among Adults with Arthritis — United States, 2017

**DOI:** 10.15585/mmwr.mm685152a1

**Published:** 2020-01-03

**Authors:** Janae D. Price, Kamil E. Barbour, Yong Liu, Hua Lu, Nancy L. Amerson, Louise B. Murphy, Charles G. Helmick, Renee M. Calanan, Michelle Sandoval-Rosario, Claudine M. Samanic, Kurt J. Greenlund, Craig W. Thomas

**Affiliations:** ^1^Division of Population Health, National Center for Chronic Disease Prevention and Health Promotion, CDC; ^2^Office of Health Promotion, Division of Chronic Disease Prevention and Control, Illinois Department of Public Health, Springfield, Illinois; ^3^Colorado Department of Public Health and Environment; ^4^Arizona Department of Health Services; ^5^Indiana State Department of Health.

An estimated 54.4 million (22.7%) U.S. adults have provider-diagnosed arthritis (arthritis), a number that is projected to rise to 78.4 million by 2040 (*1*,*2*). Chronic pain conditions like arthritis are associated with poorer mental health (*3*), especially anxiety and depression, which can impede self-care and self-management behaviors (*1*). Although the national prevalence of mental health conditions among adults with arthritis has been reported (*3*,*4*), little is known about state-specific prevalences, particularly of frequent mental distress, a useful public health measure that reflects perceived mental health status. An estimated 11.3% and 19% of U.S. adults overall have frequent mental distress and a history of depression, respectively (*5*). This analysis used 2017 Behavioral Risk Factor Surveillance System (BRFSS) data to estimate state-specific prevalence of frequent mental distress and history of depression among adults with arthritis. The median state age-adjusted prevalences of frequent mental distress and history of depression among adults with arthritis in the 50 states and the District of Columbia (DC) were 16.8% (range = 12.9% [Hawaii] to 22.4% [Kentucky]) and 32.1% (range = 17.7% [Hawaii] to 36.6% [Oklahoma]), respectively. Health care providers have an opportunity to improve the quality of life of arthritis patients by screening for mental health problems, encouraging physical activity, and making referrals to evidence-based programs such as physical activity programs,* self-management education programs^†^ (e.g., Chronic Disease Self-Management Program), psychotherapy,^§^ and cognitive behavioral therapy, that can help improve management of arthritis and mental health outcomes.

BRFSS^¶^ is a landline and cellular telephone survey conducted annually in all 50 states, DC, and U.S. territories that collects information on health-related behavioral risk factors, health care access, and chronic conditions among noninstitutionalized U.S. adults aged ≥18 years. The median survey response rate for all states and DC in 2017 was 45.8% and ranged from 30.6% (Illinois) to 64.1% (Wyoming).** For this analysis, 2017 BRFSS data were restricted to those for 147,288 adults with arthritis, defined as a “yes” response to the question “Have you ever been told by a doctor or other health care professional that you have arthritis, rheumatoid arthritis, gout, lupus, or fibromyalgia?” Frequent mental distress, a commonly used indicator of mental health, was defined as a response of ≥14 days to the question “Now thinking about your mental health, which includes stress, depression, and problems with emotions, for how many days during the past 30 days was your mental health not good?” The rationale for selecting the 14-day minimum period was based on evidence showing that clinicians and clinical researchers use a similar period as a marker for clinical depression and anxiety disorders, and a longer duration of reported symptoms is associated with a higher level of activity limitation.^††^ History of depression was defined as an affirmative response to the question “Have you ever been told you have a depressive disorder (including depression, major depression, dysthymia, or minor depression)?”

For adults with arthritis, the unadjusted, age-specific, and age-adjusted prevalences of frequent mental distress and history of depression were estimated overall, by state, and by sociodemographic characteristics. Estimates were age-adjusted using logistic regression modeling to produce predicted marginal probabilities. Differences in mental health outcomes across subgroups among adults with arthritis were tested using chi-squared tests; all differences reported were significant at α<0.05. All analyses were conducted using SAS software (version 9.4; SAS Institute) and SAS-callable SUDAAN (version 11.0.1; Research Triangle Institute) to account for the complex survey sampling design.

Overall, the nationwide unadjusted prevalence estimates of frequent mental distress and history of depression among adults with arthritis were 19.0% (95% confidence interval [CI] = 18.6–19.5) and 32.1% (95% CI = 31.5–32.6), respectively. Among adults with arthritis, the age-adjusted prevalence of frequent mental distress was significantly higher among women than among men (19.9% versus 14.6%) and persons who were lesbian/gay/bisexual compared with those who were heterosexual (28.0% versus 16.8%); it also varied by education level (Table 1). The age-adjusted prevalence of a history of depression was significantly higher among women (36.3%) than among men (24.0%), differed by race/ethnicity and education level, and was higher among lesbian/gay/bisexual adults (46.7%) than among heterosexual adults (30.5%).

**TABLE 1:** Age-specific and age-adjusted prevalence* of frequent mental distress and history of depression among U.S. adults aged ≥18 years with arthritis, by selected characteristics — Behavioral Risk Factor Surveillance System, 50 states and District of Columbia, 2017

Characteristic	No. of respondents	Weighted population (x1,000)	Unadjusted prevalence, % (95% CI)	Age-adjusted prevalence, % (95% CI)
**Arthritis and frequent mental distress**				
**Overall**	23,059	11,483	19.0 (18.6–19.5)	17.8 (17.3–18.3)
**Age group (yrs)**				
18–44	3,663	2,844	30.9 (29.3–32.5)	—
45–64	11,939	5,951	23.1 (22.3–23.8)	—
≥65	7,457	2,687	10.6 (10.1–11.2)	—
**Sex**				
Men	7,174	3,913	16.1 (15.4–16.8)	14.6 (13.9–15.3)
Women	15,874	7,566	21.0 (20.4–21.7)	19.9 (19.3–20.6)
**Race/Ethnicity**				
White, non-Hispanic	17,264	7,785	18.0 (17.5–18.5)	17.1 (16.7–17.6)
Black, non-Hispanic	2,043	1,487	21.2 (19.5–22.9)	18.9 (17.3–20.5)
Hispanic	1,506	1,235	21.8 (19.8–23.9)	19.0 (17.1–21.1)
Other/Multiracial, non-Hispanic	1,748	711	22.0 (19.1–25.1)	19.5 (16.8–22.4)
**Education level**				
Less than high school diploma	3,099	2,612	27.4 (25.8–29.1)	26.3 (24.6–28.0)
High school or equivalent	7,786	3,579	20.1 (19.3–20.9)	19.0 (18.2–19.8)
Some college	7,378	3,820	19.2 (18.3–20.0)	17.4 (16.6–18.3)
College graduate	4,737	1,431	11.1 (10.5–11.7)	10.3 (9.7–10.9)
**Sexual orientation**				
Heterosexual	9,736	6,083	17.6 (16.9–18.3)	16.8 (16.1–17.5)
Lesbian/Gay/Bisexual	581	438	33.6 (29.7–37.8)	28.0 (24.5–31.8)
**Arthritis and history of depression**				
**Overall**	43,433	19,658	32.1 (31.5–32.6)	31.3 (30.8–31.9)
**Age group (yrs)**				
18–44	5,684	4,322	46.4 (44.7–48.1)	—
45–64	20,727	9,666	37.0 (36.1–37.8)	—
≥65	17,022	5,670	21.9 (21.2–22.7)	—
**Sex**				
Men	12,391	6,244	25.3 (24.5–26.1)	24.0 (23.2–24.8)
Women	31,023	13,394	36.6 (35.9–37.4)	36.3 (35.5–37.0)
**Race/Ethnicity**				
White, non-Hispanic	34,356	13,965	31.8 (31.2–32.4)	31.5 (31.0–32.1)
Black, non-Hispanic	3,092	2,171	30.3 (28.3–32.3)	28.4 (26.4–30.4)
Hispanic	2,536	2,032	34.8 (32.5–37.1)	32.4 (30.0–34.8)
Other/Multiracial, non-Hispanic	2,693	1,158	35.5 (32.3–38.9)	33.5 (30.3–36.9)
**Education level**				
Less than high school diploma	4,712	3,797	38.5 (36.7–40.2)	38.1 (36.3–39.9)
High school or equivalent	12,998	5,633	31.1 (30.1–32.0)	30.6 (29.7–31.6)
Some college	13,885	6,789	33.7 (32.6–34.7)	32.4 (31.4–33.5)
College graduate	11,740	3,384	26.0 (25.2–26.9)	25.5 (24.6–26.4)
**Sexual orientation**				
Heterosexual	18,551	10,755	30.8 (29.9–31.6)	30.5 (29.6–31.3)
Lesbian/Gay/Bisexual	1,038	678	51.5 (47.3–55.6)	46.7 (42.5–51.0)

**Abbreviation:** CI = confidence interval.

* Estimates for all characteristics except age group were age-adjusted using logistic regression modeling to produce predicted marginal probabilities.

Age-adjusted prevalence of both mental health measures among adults with arthritis varied widely by state (Table 2). The median state age-adjusted prevalence of frequent mental distress and history of depression among adults with arthritis in all 50 states and DC was 16.8% (range = 12.9% [Hawaii] to 22.4% [Kentucky]) and 32.1% (range = 17.7% [Hawaii] to 36.6% [Oklahoma]), respectively. States with high prevalences of frequent mental distress clustered in the Appalachian and southern states, whereas a similar geographic clustering was not observed for prevalence of a history of depression (Figure).

**TABLE 2:** Age-specific and age-adjusted prevalence* of frequent mental distress and history of depression among U.S. adults aged ≥18 years with arthritis, by state — Behavioral Risk Factor Surveillance System, 50 states and District of Columbia (DC), 2017

State	Arthritis and frequent mental distress				Arthritis and history of depression			
	No. of respondents	Weighted population (x1,000)	Unadjusted prevalence, % (95% CI)	Age-adjusted prevalence, % (95% CI)	No. of respondents	Weighted population (x1,000)	Unadjusted prevalence, % (95% CI)	Age-adjusted prevalence, % (95% CI)
Alabama	526	263	22.0 (19.8–24.3)	19.7 (17.7–22.0)	892	433	35.2 (32.7–37.7)	33.5 (31.1–36.0)
Alaska	131	22	18.2 (13.9–23.5)	15.4 (11.7–20.1)	238	37	30.3 (25.4–35.8)	27.6 (23.0–32.7)
Arizona	757	237	18.7 (17.3–20.3)	17.7 (16.2–19.2)	1,430	407	31.9 (30.2–33.6)	31.4 (29.7–33.2)
Arkansas	369	159	23.2 (19.9–26.8)	20.9 (17.8–24.4)	695	262	37.6 (33.9–41.5)	35.8 (32.1–39.5)
California	333	948	16.4 (14.2–18.9)	15.6 (13.4–18.1)	692	1,789	30.6 (27.8–33.5)	30.4 (27.4–33.5)
Colorado	351	135	15.0 (13.4–16.8)	13.8 (12.2–15.5)	673	243	26.7 (24.6–28.8)	25.7 (23.7–27.8)
Connecticut	427	97	15.5 (13.8–17.4)	14.9 (13.2–16.8)	858	177	27.8 (25.7–30.1)	27.8 (25.7–30.0)
Delaware	216	34	18.2 (15.3–21.5)	16.9 (14.2–20.1)	385	62	32.8 (29.2–36.6)	32.1 (28.6–35.9)
DC	131	13	16.7 (13.8–20.0)	15.9 (13.1–19.2)	189	19	23.4 (19.9–27.3)	22.9 (19.5–26.7)
Florida	1,337	779	19.5 (17.4–21.7)	19.6 (17.4–21.9)	2,325	1,329	32.5 (30.0–35.0)	33.5 (30.9–36.2)
Georgia	269	298	17.5 (15.2–20.1)	16.0 (13.8–18.5)	480	482	27.9 (25.3–30.7)	26.6 (24.0–29.4)
Hawaii	269	32	13.7 (11.6–16.0)	12.9 (10.9–15.1)	411	42	18.3 (16.1–20.7)	17.7 (15.6–20.2)
Idaho	224	51	17.2 (14.6–20.3)	16.1 (13.6–19.0)	506	103	33.9 (30.7–37.3)	33.3 (30.0–36.6)
Illinois	233	383	16.0 (13.7–18.7)	14.7 (12.5–17.2)	436	689	28.8 (25.8–31.9)	27.8 (25.0–30.9)
Indiana	873	298	21.3 (19.8–22.9)	19.2 (17.7–20.7)	1,644	522	36.8 (35.0–38.6)	35.2 (33.4–37.0)
Iowa	307	96	16.7 (14.8–18.7)	15.7 (13.9–17.7)	678	189	32.2 (30.0–34.6)	31.9 (29.7–34.2)
Kansas	968	92	18.1 (16.8–19.4)	16.7 (15.5–18.0)	2,019	178	34.4 (32.9–35.9)	33.6 (32.1–35.1)
Kentucky	671	268	25.0 (22.5–27.7)	22.4 (20.0–25.0)	1,145	420	38.6 (35.8–41.4)	36.4 (33.7–39.2)
Louisiana	318	217	23.2 (20.5–26.1)	21.1 (18.5–23.8)	549	344	35.9 (32.9–39.0)	34.3 (31.3–37.5)
Maine	593	62	18.8 (16.8–20.9)	17.3 (15.4–19.3)	1,228	120	36.2 (33.8–38.6)	35.3 (32.8–37.8)
Maryland	689	198	17.5 (15.7–19.5)	16.2 (14.5–18.1)	1,293	324	28.4 (26.4–30.5)	27.5 (25.5–29.6)
Massachusetts	310	200	16.3 (13.7–19.4)	15.3 (12.7–18.3)	575	364	28.9 (25.6–32.4)	28.4 (25.1–31.9)
Michigan	650	466	20.3 (18.6–22.1)	18.8 (17.2–20.6)	1,262	797	34.3 (32.4–36.3)	33.3 (31.4–35.3)
Minnesota	550	115	14.1 (12.7–15.5)	13.2 (11.9–14.6)	1,234	244	29.5 (27.7–31.3)	29.1 (27.4–31.0)
Mississippi	338	152	23.9 (21.0–27.1)	21.5 (18.8–24.5)	588	227	34.7 (31.6–37.9)	32.9 (29.9–36.1)
Missouri	494	251	19.7 (17.6–22.0)	18.4 (16.4–20.6)	867	436	33.7 (31.3–36.3)	33.0 (30.5–35.6)
Montana	293	36	17.8 (15.3–20.5)	16.4 (14.0–19.0)	557	66	31.8 (28.9–34.9)	30.7 (27.8–33.8)
Nebraska	609	51	15.0 (13.4–16.8)	14.0 (12.4–15.7)	1,266	105	30.6 (28.5–32.8)	30.0 (27.9–32.2)
Nevada	189	82	18.0 (14.5–22.1)	17.1 (13.6–21.3)	298	129	28.1 (24.0–32.5)	27.6 (23.4–32.2)
New Hampshire	286	44	16.0 (13.7–18.7)	14.8 (12.6–17.3)	638	95	33.9 (31.0–36.9)	33.1 (30.2–36.1)
New Jersey	571	284	18.5 (16.3–20.9)	17.3 (15.2–19.7)	957	413	26.2 (23.8–28.8)	25.5 (23.1–28.1)
New Mexico	406	92	23.4 (20.7–26.3)	21.6 (19.1–24.4)	679	143	36.0 (33.0–39.1)	34.9 (31.9–38.0)
New York	524	587	17.6 (15.6–19.7)	16.7 (14.8–18.8)	914	907	26.4 (24.3–28.6)	26.0 (23.9–28.3)
North Carolina	297	441	23.4 (20.4–26.6)	22.0 (19.1–25.3)	476	644	33.7 (30.4–37.1)	32.9 (29.7–36.4)
North Dakota	255	21	15.2 (13.1–17.6)	13.3 (11.4–15.5)	580	44	31.7 (28.9–34.6)	29.8 (27.2–32.6)
Ohio	790	501	19.7 (17.9–21.7)	18.3 (16.6–20.1)	1,406	838	32.4 (30.3–34.5)	31.4 (29.4–33.5)
Oklahoma	437	179	22.5 (20.3–24.9)	20.3 (18.2–22.6)	805	309	38.2 (35.7–40.8)	36.6 (34.1–39.3)
Oregon	284	167	20.3 (17.9–22.9)	18.8 (16.5–21.4)	557	305	36.4 (33.6–39.2)	35.5 (32.7–38.4)
Pennsylvania	357	525	18.2 (16.0–20.7)	17.3 (15.1–19.8)	639	875	30.1 (27.5–32.9)	29.6 (27.0–32.4)
Rhode Island	319	46	20.1 (17.5–23.0)	18.5 (16.0–21.2)	652	79	34.5 (31.5–37.7)	33.3 (30.4–36.4)
South Carolina	709	223	21.4 (19.6–23.3)	20.3 (18.5–22.2)	1,257	366	34.1 (32.0–36.2)	33.7 (31.6–35.8)
South Dakota	272	23	16.5 (13.4–20.1)	15.2 (12.3–18.7)	484	41	28.6 (24.9–32.6)	27.6 (24.1–31.5)
Tennessee	429	315	21.2 (18.8–23.8)	19.2 (17.0–21.7)	772	551	36.0 (33.2–38.9)	34.6 (31.8–37.5)
Texas	618	923	21.3 (18.0–24.9)	19.7 (16.6–23.3)	1,091	1,560	35.4 (31.6–39.5)	34.6 (30.7–38.8)
Utah	359	63	15.5 (13.7–17.5)	13.7 (12.1–15.6)	835	146	35.4 (33.0–37.9)	33.9 (31.4–36.3)
Vermont	327	24	17.3 (15.2–19.6)	16.3 (14.2–18.5)	726	48	35.1 (32.5–37.8)	34.7 (32.1–37.5)
Virginia	471	272	17.2 (15.3–19.2)	15.7 (14.0–17.6)	935	497	30.8 (28.6–33.1)	29.7 (27.5–32.0)
Washington	631	246	18.5 (16.8–20.3)	16.8 (15.2–18.5)	1,381	499	36.9 (34.9–39.0)	35.7 (33.7–37.8)
West Virginia	526	130	23.6 (21.5–25.8)	21.5 (19.6–23.6)	869	200	35.8 (33.5–38.1)	34.2 (32.0–36.5)
Wisconsin	256	184	16.3 (13.9–19.1)	15.0 (12.8–17.7)	487	320	28.5 (25.6–31.5)	27.6 (24.8–30.5)
Wyoming	179	17	15.3 (12.9–18.0)	13.5 (11.4–16.1)	403	36	31.9 (28.8–35.1)	30.2 (27.2–33.4)
State median	N/A	N/A	18.2	16.8	N/A	N/A	32.5	32.1
Range	N/A	N/A	13.7–25.0	12.9–22.4	N/A	N/A	18.3–38.6	17.7–36.6

**Abbreviations:** CI = confidence interval; N/A = not applicable.

* Estimates were age-adjusted using logistic regression modeling to produce predicted marginal probabilities.

**FIGURE:**
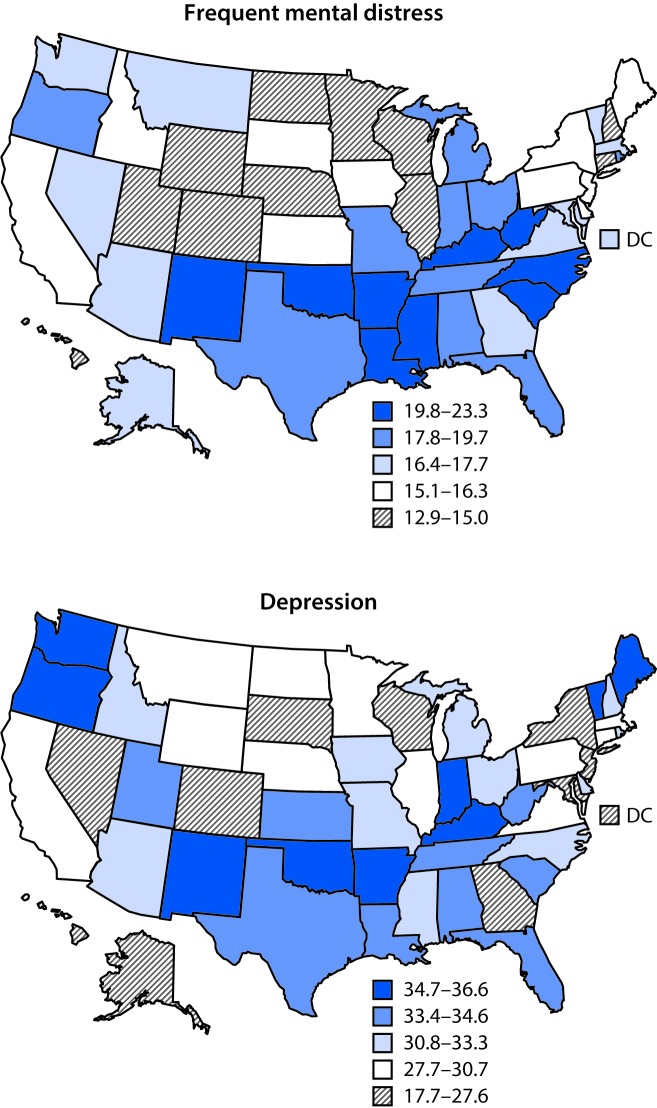
Age-adjusted prevalence* of frequent mental distress and history of depression among adults aged ≥18 years with arthritis — Behavioral Risk Factor Surveillance System, 2017 **Abbreviation:** DC = District of Columbia. * The percentage intervals for the quintile cutoffs vary because of variations in the distribution of frequent mental distress and history of depression.

## Discussion

Frequent mental distress and history of depression are common features among adults with arthritis in all states, with considerable variability across states. These findings are supported by previous studies that estimated anxiety and current depression among adults with and without arthritis (*3*,*4*). Similar to findings in an earlier report (*6*), states with high prevalences of frequent mental distress were geographically clustered, with eight of the 10 states in the highest quintile in the Appalachian and southern states. This report also provides further evidence of poorer mental health status among lesbian/gay/bisexual adults with arthritis compared with their heterosexual peers with arthritis (*4*).

A meta-analysis of 12 studies reported that persons with chronic conditions (e.g., cancer, end stage renal disease, rheumatoid arthritis, and angina) who reported current depression were three times more likely to have a reduced adherence to medical treatment recommendations (i.e., medication adherence, diet, exercise, and follow-up appointments) than were those who did not report depression (*7*). In addition, among persons with rheumatoid arthritis, symptoms of anxiety and current depression are associated with reduced response to treatment and poorer quality of life (*8*). Therefore, actively engaging adults with arthritis in evidence-based programs such as the Arthritis Self-Management Program^§§^ or the more widely available Chronic Disease Self-Management Program^¶¶^ can help address the physical and psychological needs in tandem; these programs have shown to reduce depression and improve self-efficacy in adults with arthritis (*9*). The higher prevalences of poor mental health outcomes among specific subgroups in this study, including those who are lesbian/gay/bisexual, suggests that organizations serving these persons can be important partners for promoting and increasing access to these evidence-based interventions.

The Community Preventive Services Task Force (Community Guide) recommends active screening for depression for all adults, use of trained depression care managers, and educating both patients and providers.*** Home-based supports, such as the use of community health workers, can support culturally appropriate care and further patient engagement in treatment goal-setting and self-management. Using community health workers can result in greater improvements in participant behavior and health outcomes (e.g., improvement in diabetes control) when compared with usual care.^†††^

Because of shortages in mental health care providers,^§§§^ multidisciplinary and population-based strategies that include both clinical and community approaches to addressing mental health service needs are needed for adults with arthritis. For example, allied professionals could use technology such as telemedicine in collaboration with mental health professionals, especially in rural areas (*10*) and in the delivery of care in community-based settings. The Program to Encourage Active, Rewarding Lives (PEARLS), for example, is a national evidence-based program for late-life depression that brings high quality mental health care into community-based settings that reach vulnerable older adults including those with arthritis.^¶¶¶^

The findings in this report are subject to at least five limitations. First, BRFSS data are self-reported and susceptible to recall and social desirability biases. Second, low response rates for individual states might bias findings, but sampling weights can help adjust for nonresponse bias. Third, a history of depression overestimates current depression or depressive symptoms. Fourth, the depression question does not capture adults with undiagnosed depression, and thus, might underrepresent the true proportion of respondents who are currently depressed. Finally, the arthritis question includes many types of arthritis, and prevalences of frequent mental distress and history of depression might vary among those with arthritis, rheumatoid arthritis, gout, lupus, and fibromyalgia; however, the same strategies can be used to address mental health issues for all of these conditions.

The findings from this report can be used to monitor state-specific trends in mental health among adults with arthritis. Although variation by sociodemographic and geographic characteristics exist, the prevalences of both frequent mental distress and history of depression among adults with arthritis suggests that all adults with arthritis might benefit from systematic mental health screening by their provider and, if indicated, referral to mental health services and self-management education programs and engagement with mental health and allied professionals in a variety of clinical and community settings. In addition, the use of innovative delivery models, such as employment of community health workers and telemedicine, might prove beneficial and could augment current shortages in mental health services. To further understand geographic and sociodemographic variation in characteristics among adults with arthritis, it might be beneficial to examine at the local or community level other psychosocial and access characteristics, such as employment, physical and social environmental factors, and access to social or health care services.

**Summary**

**What is already known about this topic?**

Persons with arthritis have unique challenges because the interplay between anxiety, depression, and chronic pain is cyclical, with each having the potential to exacerbate the others.

**What is added by this report?**

In 2017, frequent mental distress and history of depression were commonly reported by adults with arthritis in all states, with clustering of high prevalence of frequent mental distress in Appalachian and southern states.

**What are the implications for public health practice?**

All adults with arthritis might benefit from systematic mental health screening by their health care team (if needed, referral to mental health services) and participation in evidence-based interventions such as physical activity and self-management education programs whose proven benefits include reduced pain and improved mental health.

## Footnotes

* https://www.cdc.gov/arthritis/interventions/physical-activity.html.

† https://www.cdc.gov/arthritis/interventions/self_manage.htm.

§ https://www.nami.org/Learn-More/Treatment/Psychotherapy.

¶ https://www.cdc.gov/brfss/index.html.

** https://www.cdc.gov/brfss/annual_data/2017/pdf/2017-sdqr-508.pdf.

†† https://www.cdc.gov/hrqol/faqs.htm#10.

§§ https://www.cdc.gov/arthritis/docs/ASMP-executive-summary.pdf.

¶¶ https://www.selfmanagementresource.com/programs/.

*** https://www.thecommunityguide.org/topic/mental-health.

††† https://www.ahrq.gov/downloads/pub/evidence/pdf/comhealthwork/comhwork.pdf.

§§§
https://www.thenationalcouncil.org/wp-content/uploads/2017/03/Psychiatric-Shortage_National-Council-.pdf .

¶¶¶ https://depts.washington.edu/hprc/evidence-based-programs/pearls-program/.
